# Development and Verification of a Prognostic Ferroptosis-Related Gene Model in Triple-Negative Breast Cancer

**DOI:** 10.3389/fonc.2022.896927

**Published:** 2022-06-02

**Authors:** Song Wu, Ruilin Pan, Jibu Lu, Xiaoling Wu, Jingdong Xie, Hailin Tang, Xing Li

**Affiliations:** ^1^ State Key Laboratory of Oncology in South China, Collaborative Innovation Center of Cancer Medicine, Sun Yat-sen University Cancer Center, Guangzhou, China; ^2^ Department of Breast Surgery, The First People’s Hospital of Foshan, Foshan, China; ^3^ The First Affiliated Hospital of Zhengzhou University, Zhengzhou University, Zhengzhou, China

**Keywords:** triple-negative breast cancer (TNBC), ferroptosis, prognostic model, tumor microenvironment, mRNA signature

## Abstract

Triple-negative breast cancer (TNBC) is the subtype with the worst prognosis of breast cancer. Ferroptosis, a novel iron-dependent programmed cell death, has an increasingly important role in tumorigenesis and development. However, there is still a lack of research on the relationship between ferroptosis-related genes and the prognosis of TNBC. In this study, we obtained the gene expression profile of TNBC patients and matched clinical data from The Cancer Genome Atlas (TCGA) database. Univariate Cox analysis was used to screen out ferroptosis-related genes associated with TNBC prognosis. Then, the least absolute shrinkage and selection operator (LASSO) regression analysis was employed to establish a prognostic prediction model. A 15-ferroptosis-related gene prognostic prediction model was developed, which classified patients into low-risk (LR) or high-risk (HR) groups. Kaplan-Meier analysis results showed that the prognosis of the LR group was better. The receiver operating characteristic (ROC) curve also confirmed the satisfactory predictive ability of this model. Evaluation of the immune microenvironment of TNBC patients in the HR and LR group suggested these 15 ferroptosis-related genes might affect the prognosis of TNBC by regulating the tumor microenvironment. Our prognostic model can provide a theoretical basis for accurate prognosis prediction of TNBC in clinical practice.

## Introduction

Breast cancer is one of the malignant tumors with significant tumor heterogeneity, which can be classified into multiple subtypes according to different grading systems (1). According to the receptor expression status of breast cancer cells, those that do not express estrogen receptors (ER), progesterone receptors (PR), or HER2 are called TNBC (2). Due to the lack of effective treatments other than chemotherapy, TNBC has always been a subtype of BC with the worst prognosis (3). Although new therapies such as antibody drug conjugates (ADC), PARP inhibitors, immune checkpoint inhibitors (ICI) and other new treatment methods have been gradually used in clinical treatment with the progress of research, the overall prognosis of TNBC is still not satisfactory (4–6). TNBC, which has an incidence rate of only 12.7% of all BCs, causes 40% of BC-related mortality (7). Precise prognostic stratification is essential for the optimal choice of treatment strategies. Many factors, including age and cytomolecular genetics, are closely related to the prognosis of TNBC, but the current accurate prognostic prediction model has not yet been developed (8, 9). In summary, in order to develop a more accurate TNBC prognosis prediction model, we should determine more factors related to prognosis stratification through the analysis of abnormal cytomolecular genetics.

Ferroptosis, featuring by the massive accumulation of lipid peroxides, is a new type of programmed cell death that depends on iron (10). The mechanism of iron death is related to the imbalance of the antioxidant effect of glutathione (11). Studies have found that both iron chelators and lipophilic antioxidants can inhibit the iron death process (12, 13). Its role in tumor progression and treatment is gradually becoming a new research hotspot (14, 15). Current research has identified many genes that play a role in BC by regulating ferroptosis. More importantly, the research on iron death is mainly based on TNBC. Keiko Miyamoto et al. found that the expression level of xCT encoded by the SLC7A11 gene affects the sensitivity of TNBC to histone deacetylase inhibitors (HDACIs) by regulating ferroptosis (16). The Black Hole Quencher (BHQ)-based nanophotosensitizer complex, a ferroptosis amplifier, was developed by Mangmang Sang, et al. to inhibit the proliferation of TNBC cells *in vivo* (17). These studies indicate that ferroptosis may shed a light on the treatment of TNBC, but the relationship between ferroptosis-related genes and TNBC prognosis remains unclear.

In this study, we obtained the gene expression profile of TNBC patients and matched clinical data from The Cancer Genome Atlas (TCGA) database. Then, we developed a 15-ferroptosis-related gene pattern to stratify the TNBC prognosis by the calculated risk score, the prediction ability of which was verified. In addition, we have also explored related mechanisms through Gene Set Enrichment Analysis (GSEA) and the evaluation of immune microenvironment in high-risk (HR) and low-risk (LR) groups.

## Methods

### Data Collection

The RNA expression data of triple-negative breast cancer (TNBC) patients used in this study and the corresponding clinical data were obtained from open access databases before October 01, 2021. According to the inclusion criteria: a. Pathologically diagnosed as breast cancer; b. ER, PR, and HER2 are negative. If the HER2 level is 2+, FISH (fluorescence *in situ* hybridization) is required to be negative. We retrieved TCGA data that meet the requirements on the UCSC Xena website (18) (https://xenabrowser.net/datapages/). The “limma” R package is applied to adjust the relative gene expression level(FDR<0.05). In addition, we obtained 240 ferroptosis-related genes from the FerrDb database (19) (http://www.zhounan.org/ferrdb/legacy/index.html). All data obtained from TCGA and FerrDb is publicly available and ethical.

### Establishment of Prognostic Prediction Model

The prognostic prediction model was generated from the TCGA cohort. Firstly, we used univariate Cox analysis to identify ferroptosis-related genes (P value<0.1) which were significantly related to the prognosis of TNBC. Then, the least absolute shrinkage and selection operator (LASSO) regression analysis was used to calculate the regression coefficients of each ferroptosis-related genes. The regression coefficients are estimated with the help of 10-fold cross-validation estimator penalizes maximum likelihood estimation, and the quintessential penalty parameter λ values are determined by the minimum criteria of the penalized maximum likelihood estimator. After establishing the prognostic model, we used it to calculate the Risk Score (RS) of each patient in the TCGA cohort. The cohort was subsequently divided into high-risk and low-risk groups based on the median of RS.

### Evaluation of the Immune Microenvironment of TNBC Patients in High-Risk (HR) and Low-Risk (LR) Groups

In order to evaluate the immune microenvironment of TNBC patients in high-risk and low-risk groups, immune cell infiltration was assessed. The infiltration of immune cells, including B cells naive, B cells memory, plasma cells, T cells cd8, T cells CD4 naive, T cells CD4 memory resting, T cells CD4 memory activated, T cells follicular helper, T cells regulatory, T cells gamma delta, NK cells resting, NK cells activated, monocytes, monocytes M0, monocytes M1, monocytes M2, dendritic cells resting, dendritic cells activated, mast cells resting, mast cells activated, eosinophils, neutrophils, were assessed by CIBERSORT(https://cibersort.stanford.edu/) *p*<0.05 (20). In addition, we used ESTIMATE to calculate the Stromal Score and Immune Score of the high-risk group and the low-risk group respectively (21).

### Quantitative Real‐Time Polymerase Chain Reaction

A total of 90 RNA later‐reserved breast cancer tissue was collected from patients who diagnosed non-TNBC BC (30 patients), TNBC (30 patients) or healthy volunteers (30 person) in Sun Yat‐sen University Cancer Center. All samples were stored at −80°C until further analysis. TRIzol reagent (Thermo Fisher Scientific) was used to extract total RNA and PrimeScript™ RT Master Mix (Takara Bio) was used to transcribe RNA into cDNA. Quantitative Real‐time polymerase chain reaction was then performed using a TB Green^®^ Premix Ex Taq (Takara Bio). Student’s t‐test (two‐tailed) was used for the comparison analyses. The primers used are listed in **Table S1**.

### Functional Enrichment Analysis

After dividing the TCGA cohort into HR and LR groups based on the median risk score, GSEA v4.0.2 software was used to analyze the biological functions related to the two cohorts, using the KEGG gene set. Significant is considered as NOM p-values <0.05.

### Statistical Analysis

The predictive ability of the ferroptosis-related genes prediction model established in this study is evaluated by time-dependent Receiver Operating Characteristic (ROC) curve. In addition, the comparison of OS between the high-risk group and the low-risk group is based on Kaplan-Meier analysis. Spearman correlation R was used to analyze the relation between the ferroptosis-related genes. R (version 4.0.0) is used for all data analysis, unless otherwise stated, statistical differences are considered as p-values <0.05.

## Results

### Establishment of a Prognostic Ferroptosis-Related Gene Pattern

**Figure 1:**
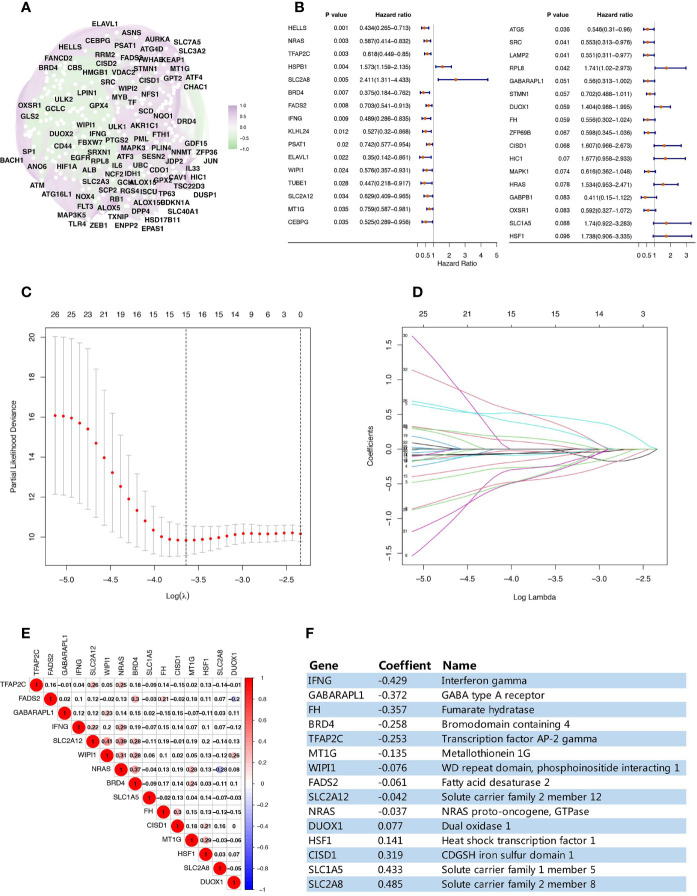
Establishment of a prognostic ferroptosis-related gene pattern. **(A)** Correlation analysis of the ferroptosis-related genes. **(B)** Forest plots interpreting the univariate Cox analysis results between ferroptosis-related genes expression and OS. **(C)** Variables filtering based on Lasso regression. **(D)** Each curve represents the change trajectory of each ferroptosis-related genes coefficient. **(E)** Corplot showing the pairwise correlation analysis of these 15 ferroptosis-related genes. **(F)** The prognostic regression model established by LASSO regression analysis.

This study analyzed a TCGA data set cohort of 158 TNBC patients, which contained complete gene expression and prognostic data (**Table S2**). We identified 240 ferroptosis-related genes in FerrDb that matched the expression data of this cohort, an overview of their correlation is shown in **Figure 1A**. Univariate Cox analysis screened out 33 ferroptosis-related genes related to TNBC overall survival (OS) (p value <0.1) (**Figure 1B**). Using LASSO regression analysis, a prognostic ferroptosis-related gene signature was constructed based on the expression of these 33 genes. Finally, a 15-ferroptosis-related gene pattern was established based on the penalized maximum likelihood estimator, with a minimum criteria optimal λ value (**Figures 1C, D**). Since these 15 genes are all related to ferroptosis, in order to rule out the multicollinearity caused by the possible correlation between them, we performed a pairwise correlation analysis on these 15 ferroptosis-related genes, which showed there was no strong correlation between them (**Figure 1E**). The formula used for Risk Score(RS) calculation was as follows: RS=-0.429 × IFNG levels - 0.372 × GABARAPL1 levels - 0.257 × FH levels - 0.258 × BRD4 levels - 0.253 × TFAP2C levels - 0.135 × MT1G levels - 0.076 × WIPI1 levels - 0.061 × FADS2 levels - 0.042 × SLC2A12 levels - 0.037 × NRAS levels + 0.077 × DUOX1 levels + 0.141 × HSF1 levels + 0.319 × CISD1 levels + 0.433 × SLC1A5 levels + 0.485 × SLC2A8 levels (**Figure 1F**).

**Figure 2:**
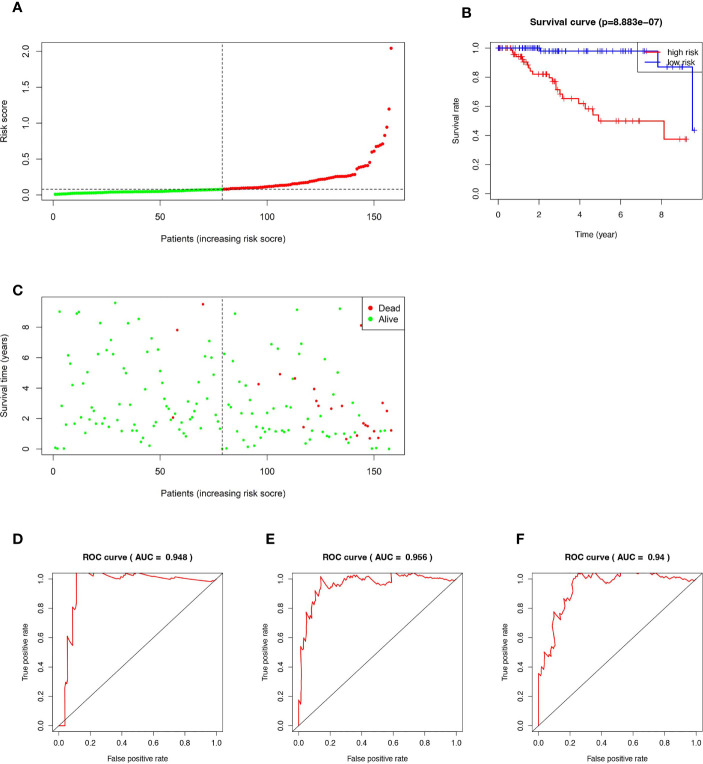
Kaplan–Meier curve, Time-dependent ROC curve, Risk score analysis and Survival status analysis of the 15-ferroptosis-related gene signature. **(A)** Risk score analysis for the 15-ferroptosis-related gene signature. **(B)** Kaplan–Meier curve of 15-ferroptosis-related gene signature in the TCGA cohort. **(C)** Survival status analysis for the 15-ferroptosis-related gene signature. **(D–F)** The time-dependent ROC curve of 1-, 3-, 5-year OS of the 15-ferroptosis-related gene signature in the TCGA cohort.

According to the established RS calculation formula, the RSs of all 158 TNBC patients were calculated respectively, then the cohort was divided into high-risk group (HR, n=79) and low-risk group (LR, n=79) based on the median of RS (**Figure 2A**). Kaplan-Meier analysis showed that the prognosis of HR patients was significantly worse than that of the LR (**Figure 2B**). As shown in **Figure 2C**, HR patients are also more likely to die prematurely, compared with LR patients. In the time-dependent ROC curve analysis, our prognostic model showed high accuracy. The AUCs for 1-, 3-, and 5 years were 0.948, 0.956, and 0.94, respectively (**Figures 2D–F**).

### Validation of the Expression Levels of the 15 Ferroptosis‐Related Genes in Clinical Specimens

**Figure 3:**
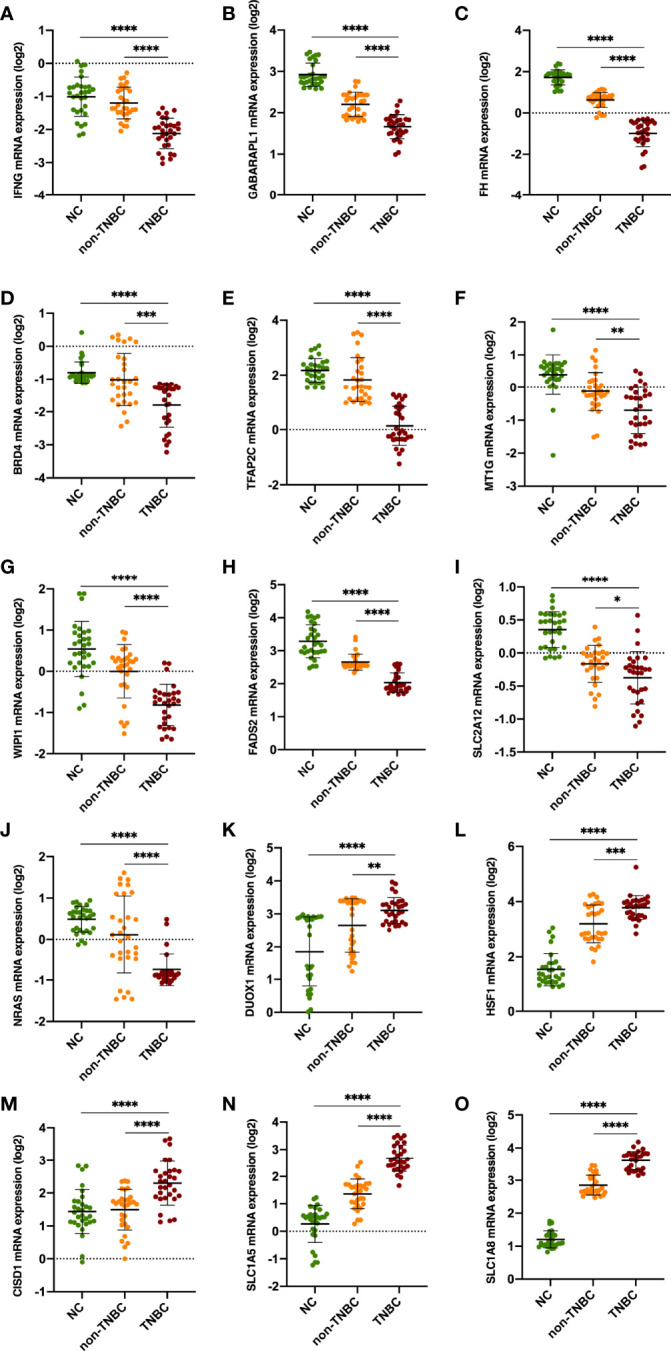
Validation of the expression levels of the 15 ferroptosis‐related genes in clinical specimens. Student’s t-test (two-tailed) was used for the comparison analyses. *P < 0.05, **P < 0.01, ***P < 0.001 ****P < 0.0001 respectively.

The expression signatures of the 15 ferroptosis‐related genes were explored in 60 BC clinical specimens. The results demonstrated that IFNG, GABARAPL1, FH, BRD4, TFAP2C, MT1G, WIPI1, FADS2, SLC2A12 and NRAS mRNA levels were downregulated in BC specimens, especially in TNBC specimens. While DUOX1, HSF1, CISD1, SLC1A5 and SLC2A8 was upregulated (**Figures 3A–O** and **Supplementary Figure 1**).

### Different Immune Microenvironment Between HR and LR TNBC Patients

**Figure 4:**
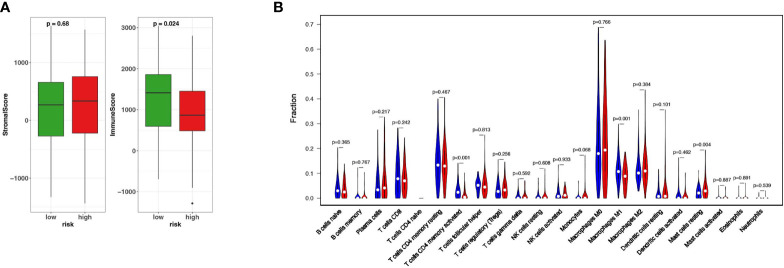
Different immune microenvironment between HR and LR TNBC patients. **(A)** The fraction of stromal and immune cells in HR and LR groups. **(B) **The Vioplot shows that the abundances of T cells CD4 memory activated, macrophages M1 (P < 0.01), and mast cells resting(P < 0.05) were significantly different between HR and LR.

The important role of tumor immune microenvironment in tumorigenesis and development has been confirmed by more and more studies (3). In order to confirm whether there is a difference in the immune microenvironment between HR patients and LR patients, firstly we used ESTIMATE to calculate the Stromal Score and Immune Score of the HR and LR patients respectively. The results showed that the Immune Score of the two groups was statistically different (P<0.05) while the Stromal Score of them are not (**Figure 4A**). In order to further investigate the infiltration of various immune cells in the two groups, we used CIBERSORT to detect the abundance of various immune cells in the two groups. The results showed that the abundances of T cells CD4 memory activated (P<0.001), macrophages M1 (P<0.01), and mast cells resting(P<0.05) were significantly different in two groups (**Figure 4B**). These results indicate that these 15 ferroptosis-related genes may affect the prognosis of TNBC by regulating the tumor microenvironment.

### Functional Enrichment Analysis Associated With Ferroptosis-Related Genes

**Figure 5:**
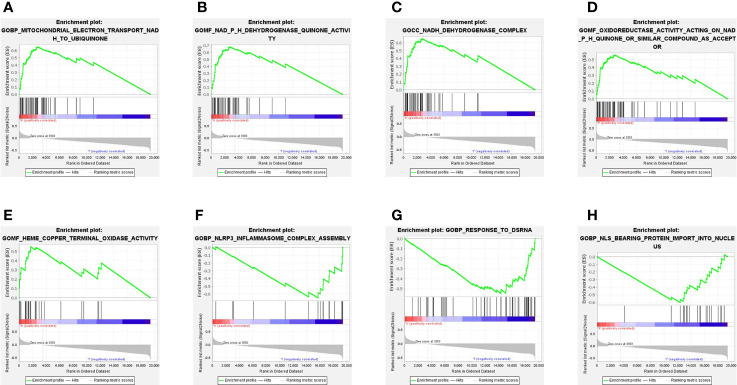
Gene set enrichment analysis (GSEA) in the TCGA cohort. **(A–E)** Signaling pathways significantly enriched in HR group. **(F–H)** 3 non-ferroptosis-related pathways enriched in LR group.

To explore the biological functions and networks associated with risk score, we performed GSEA in the TCGA cohort. As we expected, because the risk score was calculated based on a 15-ferroptosis-related gene pattern, the 5 significant enrichment pathways detected in the HR group were all related to ferroptosis. These pathways are mainly related to the oxidase activity and electron transfer process in mitochondria (**Figures 5A–E**). Specifically, in addition to the ferroptosis-related pathways, we found the enrichment of other pathways. Among these pathways were inflammasome complex assembly, response to dsRNA and protein import into nucleus (**Figures 5F–H**).

## Discussion

Breast cancer is the most common malignant tumor among Chinese women. With the increasing incidence, breast cancer has become the most common cause of cancer-related death in women (22). Due to tumor heterogeneity and the long-term lack of effective therapies other than chemotherapy, triple-negative breast cancer (TNBC) has the worst prognosis of all BC subtypes (23–25). Therefore, studying the molecular mechanism of TNBC and searching for new prognostic indicators can help improve the survival of breast cancer patients. Ferroptosis is a novel programmed cell death that depends on iron, characterized by the accumulation of lipid peroxides (10). With the progress of related research, it has gradually become a promising treatment for cancer (26). A latest study by Y. Wen et al. confirmed the existence of ferroptosis in BC. The ferroptosis inducer 18-β-glycyrrhetinic acid (GA) could trigger ferroptosis in TNBC cells by inducing the production of ROS/RNS, which accelerated the accumulation of peroxidation and the death of TNBC cells (27). In addition, many studies have found that several genes regulate ferroptosis in TNBC (28–30). Nevertheless, the relationship between ferroptosis-related genes and TNBC prognosis is still lacking in research.

Herein, we obtained ferroptosis-related genes from a manually curated database for ferroptosis (19). 33 ferroptosis-related genes related to TNBC OS were screened out from them by univariate Cox analyses, which were subsequently used to generate a 15-ferroptosis-related gene pattern in the TCGA-BRCA cohort. Using this prognostic prediction model, patients were classified into HR or LR groups based on the median of risk score. Kaplan-Meier analysis results showed that the prognosis of the LR group was better. The receiver operating characteristic (ROC) curve also confirmed that the model had satisfactory predictive ability.

The 15 ferroptosis-related genes (NRAS, TFAP2C, SLC2A8, BRD4, FADS2, IFNG, WIPI1, SLC2A12, MT1G, GABARAPL1, DUOX1, FH, CISD1, SLC1A5, HSF1), which were included in the prognostic prediction model, most have been reported to regulate tumor progression through pathways such as cell energy metabolism, protein transport, and immune response etc. NRAS is an oncogene which encodes a GTPase. It has been confirmed to be associated with a variety of tumors including juvenile myelomonocytic leukemia, follicular thyroid cancer and somatic rectal cancer (31). Transcription factor AP-2 gamma(TFAP2C) is a transcription factors shaping ferroptosis sensitivity, which provided novel insights into cancer therapy (32). SLC2A8 and SLC2A12, also known as GLUT8/GLUT12, are members of Glut (SLC2A) family of membrane transport proteins. It is highly expressed in a variety of tumor cells and provides glucose necessary for survival, growth and proliferation of cancer cells (33, 34). SLC1A5, which belongs to solute carrier family 1 (SLC1A), regulates the progression of several tumors (35, 36). BRD4 is involved in regulating the expression of multiple oncogenes and the assembly process of super-enhancers, which is a member of the Bromodomain and Extraterminal (BET) protein family (37). The FADS2 gene can regulate the unsaturation of fatty acids by synthesizing double bonds between carbon atoms (38). IFNG (interferon gamma) plays an important role in tumor immunotherapy. How to avoid adaptive immune resistance by blocking the IFING signal pathway is a research hotspot in immunotherapy (39, 40). WIPI1 has been confirmed by many studies as a key molecule in the process of autophagy (41, 42). MT1G is one of the eight functional isoforms known to MT1, one of the four subtypes of metallothioneins (MTs), plays an important role in oxidative stress (43). GABARAPL1(GEC1) was originally found to be responsible for the intracellular transport of proteins. Recently studies have found that it is also implicated in tumor progression, proliferation, autophagy and various mechanisms (44). DUOX1 is a NADPH oxidase catalyzing the accumulation of hydrogen peroxide (45). Fumarase(FH) is an essential dehydratase of the citric acid cycle in the mitochondria, which regulates ferroptosis through modulation of energy metabolism (46). CISD1 has been confirm to be a ferroptosis inhibitors which ferroptosis by protection against lipid peroxidation of mitochondria (47). Heat shock factor 1 (HSF1) is a key transcription factor of HSP, and its important regulatory role in tumors relies on its protective role of guaranteeing proper folding and distribution of Intracellular proteins (48). But the relationship between ferroptosis-related genes and TNBC prognosis needs further investigation.

Our prognostic model mainly reflects the internal characteristics of TNBC cells. In order to explore its connection with the external characteristics of tumor microenvironment, we used the Cibersort algorithm and ESTIMATE to evaluate the immune microenvironment of TCGA-BRCA cohort (48, 49). We confirmed the differences of immune microenvironment of TNBC patients between HR and LR groups. The fraction of immune cells is higher in the LR group, and the abundances of T cells CD4 memory activated, macrophages M1 is also higher than the HR group. However, the GSEA analysis did not identified immune-related pathways and biological processes enriched in HR or LR groups. These results indicate that whether the alterations of ferroptosis-related genes shape the immune contexture is worthy of further study.

Although our prognostic model exhibits satisfactory predictive ability, there are still several limitations. First of all, our prognostic model was based on the 158 TCGA-BRCA cohort. Hence, additional validation of its predictive power is needed in an external cohort. In addition, the relationship between immune microenvironment and the riskscore deserves further exploration in follow-up studies.

## Conclusions

We systematically analyzed the TNBC cell-intrinsic features of ferroptosis in TCGA database and developed a 15 ferroptosis-related genes prognostic model. The satisfactory accuracy in predicting OS of our prognostic model was demonstrated, which could possibly be used as an indicator of TNBC prognosis. Our prognostic model can provide a theoretical basis for accurate prognosis prediction of TNBC in clinical practice.

## Data Availability Statement

Publicly available datasets were analyzed in this study. This data can be found here: UCSC Xena website (https://xenabrowser.net/datapages/) and FerrDb (http://www.zhounan.org/ferrdb/legacy/index.html).

## Ethics Statement

The studies involving human participants were reviewed and approved by Sun Yat-sen University Cancer Center. The patients/participants provided their written informed consent to participate in this study.

## Author Contributions

Conception and design: SW and RP; (II) Administrative support: HT; (III) Provision of study materials or patients: XL; (IV) Collection and assembly of data: SW and XW; (V) Data analysis and interpretation: SW and JX; (VI) Manuscript writing: All authors; (VII) Final approval of manuscript: All authors.

## Funding

This study was supported by the National Natural Science Foundation of China (Nos. 81772961, HT).

## Conflict of Interest

The authors declare that the research was conducted in the absence of any commercial or financial relationships that could be construed as a potential conflict of interest.

## Publisher’s Note

All claims expressed in this article are solely those of the authors and do not necessarily represent those of their affiliated organizations, or those of the publisher, the editors and the reviewers. Any product that may be evaluated in this article, or claim that may be made by its manufacturer, is not guaranteed or endorsed by the publisher.

## Supplementary Material

Supplementary Figure 1 Validation of the expression levels of the 15 ferroptosis‐related genes in clinical specimens. Student’s t-test (two-tailed) was used for the comparison analyses. ** P<0.01, ***P<0.001 ****P<0.0001 respectively.
