# Supplementary figures and images for: Development and Verification of a Prognostic Ferroptosis-Related Gene Model in Triple-Negative Breast Cancer
Source: Front Oncol. 2022 Jun 2;12:896927. doi: 10.3389/fonc.2022.896927 (PMC9202593; doi:10.3389/fonc.2022.896927)

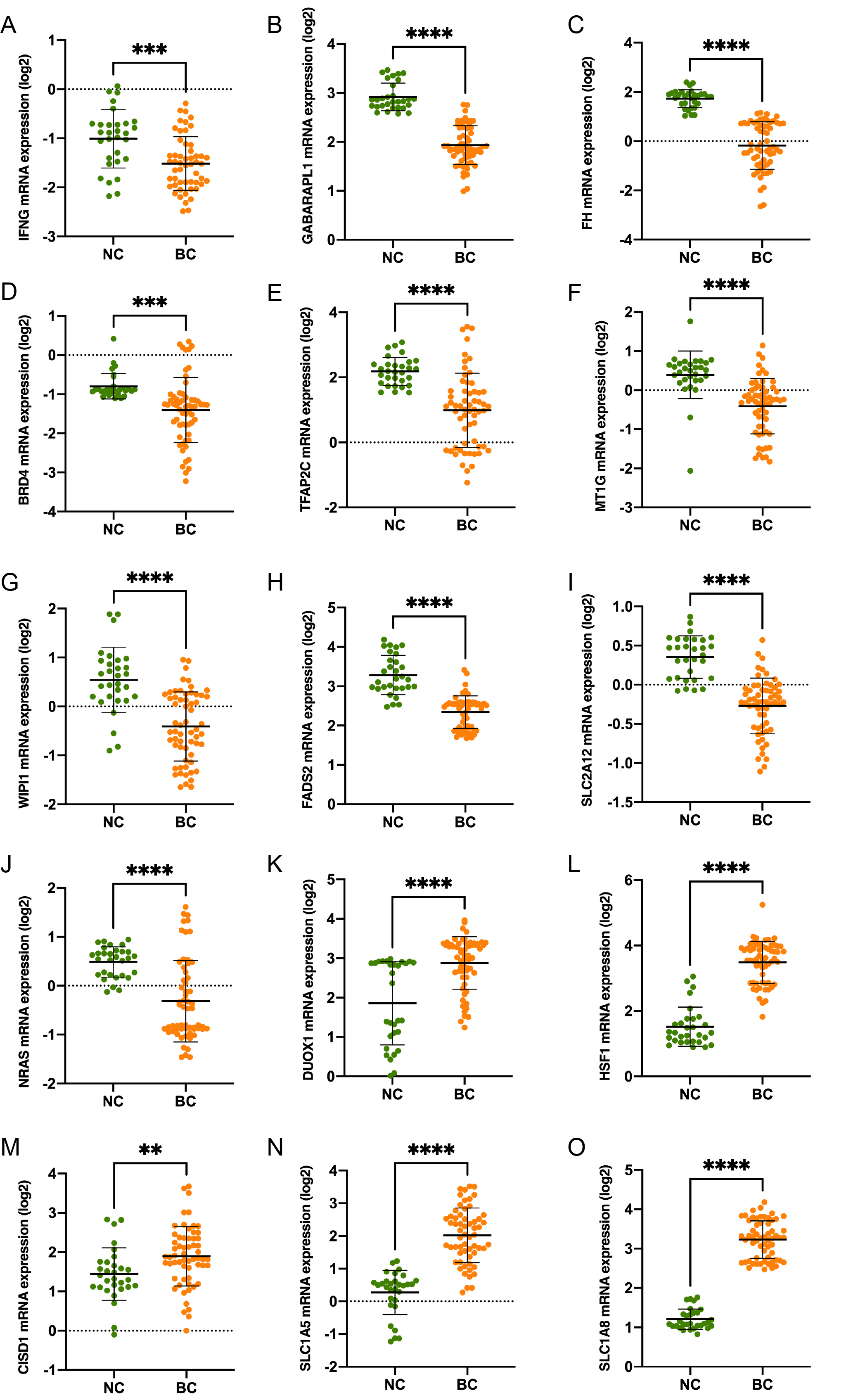

Supplement: Supplementary Figure 1 — Validation of the expression levels of the 15 ferroptosis‐related genes in clinical specimens. Student’s t-test (two-tailed) was used for the comparison analyses. ** P<0.01, ***P<0.001 ****P<0.0001 respectively. [file Image_1.jpeg]
