# Supplementary material for: Development and Verification of a Prognostic Ferroptosis-Related Gene Model in Triple-Negative Breast Cancer
Source: Front Oncol. 2022 Jun 2;12:896927. doi: 10.3389/fonc.2022.896927 (PMC9202593; doi:10.3389/fonc.2022.896927)
Supplement: Supplementary file 2 [file Table_1.docx]

| *IFNG* | Forward Primer | TCGGTAACTGACTTGAATGTCCA |
| --- | --- | --- |
|  | Reverse Primer | TCGCTTCCCTGTTTTAGCTGC |
| *GABARAPL1* | Forward Primer | ATGAAGTTCCAGTACAAGGAGGA |
|  | Reverse Primer | GCTTTTGGAGCCTTCTCTACAAT |
| *FH* | Forward Primer | GGAGGTGTGACAGAACGCAT |
|  | Reverse Primer | CATCTGCTGCCTTCATTATTGC |
| *BRD4* | Forward Primer | ACCTCCAACCCTAACAAGCC |
|  | Reverse Primer | TTTCCATAGTGTCTTGAGCACC |
| *TFAP2C* | Forward Primer | CTGTTGCTGCACGATCAGACA |
|  | Reverse Primer | CTCAGTGGGGTTCATTACGGC |
| *MT1G* | Forward Primer | AAAGGGGCATCGGAGAAGTG |
|  | Reverse Primer | GCAAAGGGGTCAAGATTGTAGC |
| *WIPI1* | Forward Primer | ACTAAAGCCGGGTATAAGCTGT |
|  | Reverse Primer | CGGGATTTCATTGCTTCCGTG |
| *FADS2* | Forward Primer | TGACCGCAAGGTTTACAACAT |
|  | Reverse Primer | AGGCATCCGTTGCATCTTCTC |
| *SLC2A12* | Forward Primer | GAGGCTGCGGCATGTTTAC |
|  | Reverse Primer | CCAAGTTCATAACCCACCAGG |
| *NRAS* | Forward Primer | ATGACTGAGTACAAACTGGTGGT |
|  | Reverse Primer | CATGTATTGGTCTCTCATGGCAC |
| *DUOX1* | Forward Primer | CCTGGCTCTAGCATGGACAC |
|  | Reverse Primer | CTGCACCTCCCACGAAATG |
| *CISD1* | Forward Primer | CCTTCACATCCAGAAAGACAACC |
|  | Reverse Primer | CTCTTCGTTATGTTTTGTGTGAGC |
| *SLC1A5* | Forward Primer | GAGCTGCTTATCCGCTTCTTC |
|  | Reverse Primer | GGGGCGTACCACATGATCC |
| *SLC2A8* | Forward Primer | CTAGTGGCCCCGGTCTACAT |
|  | Reverse Primer | CCGACGACGACCATTAGCTG |

**Supplementary Table 1** | qRT-PCR Primers for 15 ferroptosis-related genes
