# Supplementary material for: Development and Verification of a Prognostic Ferroptosis-Related Gene Model in Triple-Negative Breast Cancer
Source: Front Oncol. 2022 Jun 2;12:896927. doi: 10.3389/fonc.2022.896927 (PMC9202593; doi:10.3389/fonc.2022.896927)
Supplement: Supplementary file 4 [file Table_3.docx]

| **Symbol** | **Name** | **Type** |
| --- | --- | --- |
| *IFNG* | Interferon gamma | driver |
| *GABARAPL1* | GABA type A receptor associated protein like 1 | driver |
| *FH* | Fumarate hydratase | suppressor |
| *BRD4* | Bromodomain containing 4 | suppressor |
| *TFAP2C* | Transcription factor AP-2 gamma | marker |
| *MT1G* | Metallothionein 1G | suppressor |
| *WIPI1* | WD repeat domain, phosphoinositide interacting 1 | driver |
| *FADS2* | Fatty acid desaturase 2 | suppressor |
| *SLC2A12* | Solute carrier family 2 member 12 | marker |
| *NRAS* | NRAS proto-oncogene, GTPase | driver |
| *DUOX1* | Dual oxidase 1 | driver |
| *CISD1* | CDGSH iron sulfur domain 1 | suppressor |
| *SLC1A5* | Solute carrier family 1 member 5 | driver |
| *SLC2A8* | Solute carrier family 2 member 8 | marker |

**Supplementary Table 3** | 15 ferroptosis-related genes

*Drivers are genes that promote ferroptosis; Suppressors are genes that prevent ferroptosis; Markers are genes that indicate the occurrence of ferroptosis.
